# Low Serum BAFF Concentration Is Associated with Response to TNF Inhibitors in Seropositive Patients with Rheumatoid Arthritis

**DOI:** 10.3390/jcm11175207

**Published:** 2022-09-02

**Authors:** Borja Hernández-Breijo, Ioannis Parodis, Marta Novella-Navarro, Ana Martínez-Feito, Victoria Navarro-Compán, Mariana Díaz-Almirón, Dora Pascual-Salcedo, Alejandro Balsa, Chamaida Plasencia-Rodríguez

**Affiliations:** 1 Immuno-Rheumatology Research Group, Hospital La Paz Institute for Health Research-IdiPAZ, 28046 Madrid, Spain; 2 Division of Rheumatology, Department of Medicine Solna, Karolinska Institutet, 17176 Stockholm, Sweden; 3 Department of Gastroenterology, Dermatology and Rheumatology, Karolinska University Hospital, 17176 Stockholm, Sweden; 4 Department of Rheumatology, Faculty of Medicine and Health, Örebro University, 70182 Örebro, Sweden; 5 Rheumatology, La Paz University Hospital, 28046 Madrid, Spain; 6 Immunology Unit, La Paz University Hospital, 28046 Madrid, Spain; 7 Biostatistics Unit, Hospital La Paz Institute for Health Research-IdiPAZ, 28046 Madrid, Spain

**Keywords:** rheumatoid arthritis, BAFF, B cells, TNF inhibitors, biologics, biomarkers, autoimmune diseases, autoantibodies

## Abstract

We investigated B-cell-activating factor (BAFF) in relation to response to treatment with TNF inhibitors (TNFis) in rheumatoid arthritis (RA). This was a longitudinal study including 158 patients with RA treated with TNFis and followed up for 6 months. Clinical response at 6 months of treatment was defined according to the EULAR criteria for good responders (GRs). BAFF concentration was measured in serum samples, collected at baseline and at 6 months. Associations with EULAR response were evaluated using univariable and multivariable logistic regression models. ROC analysis was performed to determine the optimal threshold of serum BAFF concentration associated with good EULAR response to treatment. After 6 months of TNFi treatment, 24% of patients were GRs. They had a lower BMI, lower baseline DAS28 and lower baseline serum BAFF concentration than non-responders. After 6 months of TNFi treatment, autoantibody-positive patients who attained GR had significantly lower serum BAFF concentrations compared with patients who did not. Serum BAFF < 968 pg/mL at 6 months represented the concentration likely to best discriminate between GR and non-GR at 6 months of TNFi treatment. Autoantibody-seropositive patients who had serum BAFF < 968 pg/mL at 6 months demonstrated a more than four-fold increased probability to be GRs compared with patients with higher BAFF concentrations. In conclusion, serum BAFF concentrations were associated with response to TNFis in seropositive RA patients, corroborating the importance of the B-cell compartment in RA.

## 1. Introduction

Tumour necrosis factor inhibitors (TNFis) are widely used for the treatment of patients with rheumatoid arthritis (RA) who do not respond to conventional synthetic disease-modifying anti-rheumatic drugs (csDMARDs). However, 20–40% of patients do not achieve an adequate clinical response to TNFis [1]. Despite continuous advances in the understanding of the molecular mechanisms in relation to responses to TNFi therapy, there is still a lack of objective parameters that have been proven to be associated with clinical response to TNFis in RA [2,3].

The latest breakthroughs in the physiopathology of RA have highlighted the activation of B cells as a trigger of the joint flare initiation. Thus, B cells emerged as central in the pathogenesis of the disease [4,5]. The B-cell-activating factor (BAFF) is a cytokine that belongs to the TNF ligand superfamily and is primarily produced by monocytes, dendritic cells and neutrophils. BAFF is essential for B-cell activation, differentiation and survival. These effects are mediated by the interaction between BAFF and its cell surface receptors (BAFF-R, TACI and BCMA), which activate the NF-κB signalling pathway to further trigger essential effector signals for the formation and maintenance of B cells [6].

Several investigations have pointed out the role of BAFF in the development of autoantibodies, such as rheumatoid factor (RF) and anti-citrullinated protein antibodies (ACPAs) [7,8,9,10]. The presence of these autoantibodies in RA patients would imply a higher degree of inflammation during disease, which results in a more aggressive and joint-erosive condition for the patients showing higher disease activity, as well as worse prognosis owing to accelerated progression of the disease [11,12,13].

The main objective of this study was to investigate the role of BAFF in the clinical response to treatment with TNFis in patients with RA, stratified by autoantibody status.

## 2. Materials and Methods

### 2.1. Patients

For this study, 158 patients with RA from the RA-Paz cohort were included. The RA-Paz cohort is a prospective, observational cohort comprising patients with RA who have initiated biological DMARD treatment [14]. All enrolled patients fulfilled the ACR/EULAR 2010 classification criteria for RA [15], were over 18 years, had a moderate or high disease activity (DAS28 > 3.2) and fulfilled the criteria of the Spanish Society of Rheumatology recommendations regarding the use of biological therapies in RA [16]. Clinical data were systematically collected in a database by means of an electronic CRF at the Biologic Unit of La Paz University Hospital, and serum samples were frozen immediately after collection and stored in the biobank of the Hospital.

Disease activity was assessed using the Disease Activity Score 28 (DAS28) at baseline (at the time of TNFi initiation) and at 6 months of treatment. Clinical response at 6 months of treatment was defined according to the European Alliance of Associations for Rheumatology (EULAR) criteria for good responders: DAS28 < 3.2 and ∆DAS28 > 1.2 [17]. The patients were treated with TNFis (infliximab, adalimumab, certolizumab pegol, golimumab or etanercept) and followed up for 6 months. Serum samples were collected at baseline and at 6 months within a maximum of 24 h before the drug administration of subcutaneous TNFis, or immediately before intravenous infliximab infusions. BAFF levels were measured in stored serum samples from baseline and at 6 months.

### 2.2. Measurement of the Serum Concentration of BAFF, RF and ACPA

Serum BAFF concentrations were measured at the baseline and at 6 months of TNFi treatment using the BAFF Human Instant ELISA Kit (R&D Systems, Minneapolis, MN, USA) following the manufacturer’s instructions. Serum RF concentrations were measured by nephelometry using the BNII System and N Latex RF Kit (Siemens Healthcare Diagnostics Inc., Newark, DE, USA) according to the instruction manual. Serum ACPA was determined by ELISA as anti-CCP2 with the Immunoscan CCPlus kit (Svar, Malmö, Sweden).

### 2.3. Statistical Analyses

Descriptive analyses were performed for the demographic and clinical variables. The results are shown as mean and standard deviation (SD) or median (interquartile range, IQR) depending on data distribution for continuous variables, and relative frequencies for categorical variables. The frequency data were compared using the Pearson’s chi-square or Fisher’s exact tests. Comparisons of unpaired continuous data were conducted using the unpaired *t*-test or Mann–Whitney U test, depending on data distribution. Comparisons of paired continuous data were conducted using the paired *t*-test or Wilcoxon signed-rank test, depending on data distribution.

Associations between the EULAR response at 6 months and clinical/serological variables were evaluated using univariable and multivariable logistic regression models, and data are presented as odds ratios (ORs) and 95% confidence intervals (CIs). Any variable having a *p*-value < 0.1 at the univariate test was selected for the multivariate analysis. The presence of interactions between covariates was tested, and stratifications were performed for significant interactions (*p* < 0.05). In the case of no interaction, the model was later adjusted for these covariates. Finally, receiver operating characteristic (ROC) analysis was performed to determine the baseline serum BAFF concentration that is more likely associated with EULAR response at 6 months of TNFi treatment in seropositive patients. Only this group was selected based on differences observed in univariate and multivariate analysis. The optimal threshold was determined as the highest Youden index.

The correlated BAFF concentration data were fitted with a generalised estimating equation (GEE) model with normal distribution, identity link function and exchangeable work correlation matrix (EWCM). The response variable means were estimated and compared by least squares between the different levels of each categorical independent variable. The overall effect of each continuous independent variable was analysed using the type III test. All tests were considered bilateral with 95% confidence interval. The software used for this purpose was SAS Enterprise 8.2. The Statistical Package for the Social Sciences (SPSS, Chicago, IL, USA) version 24 was used for all the other analyses. *p*-value < 0.05 was considered statistically significant. The GraphPad Prism version 9 (GraphPad Software, San Diego, CA, USA) was used to prepare the graphs.

## 3. Results

### 3.1. Patient and Disease Characteristics

A total of 158 patients with RA for whom TNFi treatment was initiated (72 infliximab, 33 certolizumab pegol, 24 adalimumab, 15 etanercept and 14 golimumab) were included in this study. Demographics and clinical characteristics are summarised in Table 1. Overall, 38 patients (24%) attained good EULAR response (GR) at 6 months of treatment. These patients had significantly lower body mass index (BMI) (*p* = 0.02) and lower baseline DAS28 (*p* = 0.02) compared with patients who did not attain GR. In addition, patients who attained GR showed lower baseline serum BAFF concentrations (*p* = 0.04).

**Figure 1:**
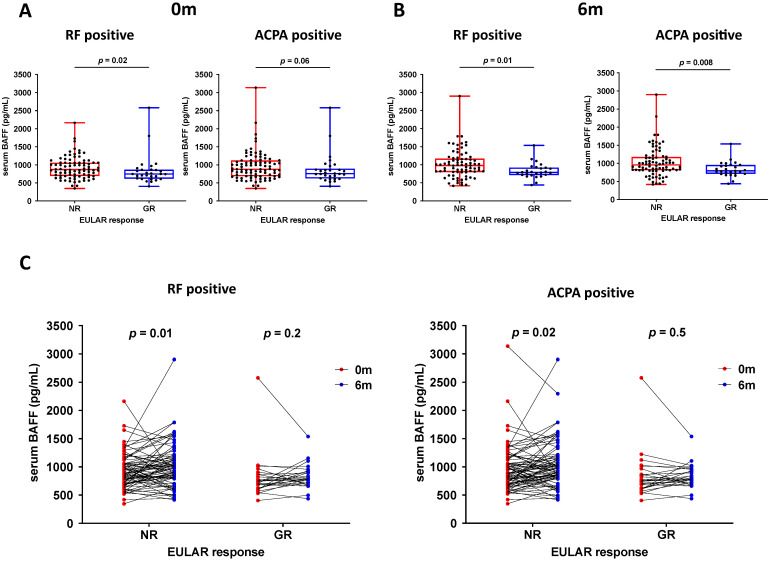
Serum BAFF concentrations (median [min–max]) stratified by EULAR response at the baseline and at 6 months of TNFi treatment in RF- (**A**) or ACPA-seropositive patients (**B**). (**C**) Change in BAFF concentrations during 6 months of TNFi treatment in RF or ACPA-seropositive patients, in relation to EULAR response. *p*-value < 0.05 was considered statistically significant. BAFF, B-cell-activating factor; RF, rheumatoid factor; ACPA, anti-citrullinated protein antibody; NR, non-responders; GR, good responders.

In order to elucidate the role of BAFF in the disease considering its involvement in autoantibody formation, the analysis was stratified according to autoantibody positivity (RF status: 32 RF- and 126 RF+; ACPA status: 24 ACPA- and 134 ACPA+; Table 1). Baseline DAS28 was lower in seropositive patients who attained GR (mean ± SD; RF+: 5.0 ± 0.6 for GR vs. 5.5 ± 1.4 for non-GR, *p* = 0.02; ACPA+: 4.9 ± 0.8 for GR vs. 5.4 ± 1.4 for non-GR, *p* = 0.02). Baseline BAFF concentrations were lower in both RF- and ACPA-positive patients who attained GR. However, this reached statistical significance only in RF-positive patients (median [IQR]: 746 [616–865] pg/mL for GR vs. 870 [691–1060] pg/mL for non-GR, *p* = 0.02) (Figure 1A).

### 3.2. Association of BAFF Concentrations and Patient Characteristics with EULAR Response at 6 Months of TNFi Treatment in Seropositive Patients

Regardless of the predominant autoantibody (RF or ACPA), we found that serum BAFF concentration at 6 months of TNFi treatment was significantly lower in seropositive patients who attained GR compared with seropositive patients who did not attain GR (RF+: 787 [715–922] pg/mL for GR vs. 980 [795–1170] pg/mL for non-GR, *p* = 0.01; ACPA+: 793 [712–956] pg/mL for GR vs. 955 [808–1176] pg/mL for non-GR, *p* = 0.008) (Figure 1B). However, there were no differences in seronegative patients (RF-: 845 [713–1011] pg/mL for GR vs. 846 [670–1105] pg/mL for non-GR, *p* = 0.9; ACPA-: 856 [697–992] pg/mL for GR vs. 758 [715–1090] pg/mL for non-GR, *p* = 0.8). Therefore, the association between BAFF concentrations and clinical response was evaluated only in autoantibody-positive patients.

In addition, we analysed whether TNFi treatment modulates BAFF concentration in these patients. The median serum BAFF concentration was significantly increased after 6 months of TNFi treatment in non-GRs, regardless of the predominant autoantibody (RF+; 0 month: 870 [691–1060] pg/mL, 6 months: 980 [795–1170] pg/mL, *p* = 0.01//ACPA+; 0 month: 876 [684–1120] pg/mL, 6 months: 955 [808–1176] pg/mL, *p* = 0.02). On the contrary, this modulation was not found among patients who achieved GR (RF+; 0 month: 746 [616–865] pg/mL, 6 months: 787 [715–922] pg/mL, *p* = 0.2//ACPA+; 0 month: 754 [622–891] pg/mL, 6 months: 793 [712–956] pg/mL, *p* = 0.5) (Figure 1C). In order to investigate whether changes in serum BAFF concentrations between the baseline and 6 months were influenced by factors with confounding potentiality (included in Table 1), a GEE model was constructed; none among those variables were independently associated with the change in serum BAFF concentrations during the 6-month follow-up (Table A1).

**Figure 2:**
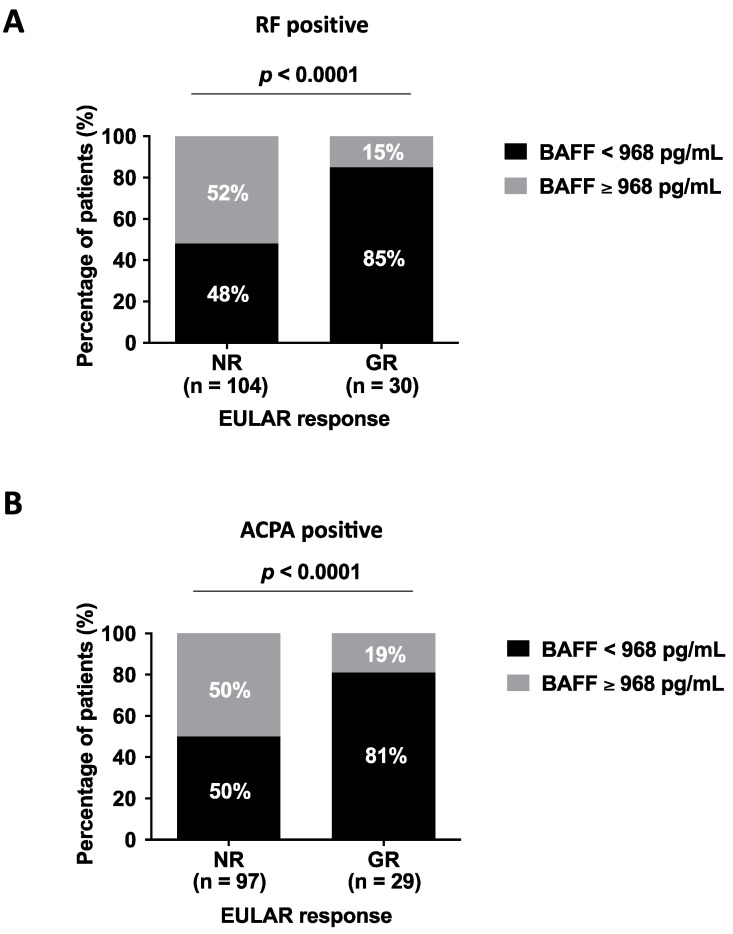
Application of the serum BAFF concentration threshold to separate EULAR good responders from non-responders at 6 months of TNFi treatment in RF- (**A**) or ACPA-seropositive patients (**B**). *p*-value < 0.05 was considered statistically significant. BAFF, B-cell-activating factor; RF, rheumatoid factor; ACPA, anti-citrullinated protein antibody; NR, non-responders; GR, good responders.

In order to determine an optimal threshold value for serum BAFF concentrations associated with GR at 6 months of TNFi treatment, ROC analyses were employed. The AUC among seropositive patients was similar, regardless of the predominant autoantibody (RF+: AUC = 0.67, 95% confidence interval: 0.56–0.78, *p* = 0.01; ACPA+: AUC = 0.67, 95% confidence interval: 0.56–0.78, *p* = 0.009). It was found, for both cases, that serum BAFF concentrations at 6 months lower than 968 pg/mL represented concentrations that were associated with GR at 6 months of TNFi treatment, with a positive likelihood ratio of 2.7. Out of the 29 RF-positive patients who attained GR, 85% had serum BAFF at 6 months below 968 pg/mL vs. 15% above before (*p* < 0.0001) (Figure 2A). Similarly, out of the 30 ACPA-positive patients who attained GR, 81% had serum BAFF at 6 months below 968 pg/mL vs. 19% above before (*p* < 0.0001) (Figure 2B).

According to the results of the univariable regression analysis of RF-positive patients, serum BAFF < 968 pg/mL at 6 months (OR: 5.95; 95% CI: 1.87–18.88) was associated with GR. Moreover, a shorter disease duration (OR: 0.93; 95% CI: 0.86–0.99), a lower BMI (OR: 0.92; 95% CI: 0.84–1.00) and lower baseline DAS28 (OR: 0.74; 95% CI: 0.54–1.03) tended to be associated with GR, yielding *p*-values < 0.1 (Table 2). Therefore, these variables were included as covariates in further statistical analyses. Next, a multivariable analysis including patient and disease characteristics with a *p*-value < 0.1 in the univariable analysis (disease duration, ACPA positivity, BMI and DAS28) was performed. This revealed that serum BAFF < 968 pg/mL at 6 months (OR: 7.94; 95% CI: 2.32–27.22) remained significantly and independently associated with GR (Table 2).

In addition, similar analyses were performed for ACPA-positive patients. The univariable analysis revealed that serum BAFF < 968 pg/mL at 6 months (OR: 4.40; 95% CI: 1.52–12.77) was significantly associated with GR. In this case, although non-significant, a lower BMI (OR: 0.92; 95% CI: 0.84–1.01) and lower baseline DAS28 (OR: 0.74; 95% CI: 0.53–1.04) tended to be associated with GR, yielding *p*-values < 0.1 (Table 2). Next, a multivariable analysis was performed. This revealed that serum BAFF < 968 pg/mL at 6 months (OR: 4.74; 95% CI: 1.58–14.23) remained independently associated with GR in seropositive patients (Table 2).

## 4. Discussion

In this study, we aimed to investigate the association between serum BAFF concentrations and clinical response to TNFis in patients with RA. We found that both RF- and ACPA-positive patients who attained good EULAR response had lower serum BAFF concentrations at 6 months of TNFi treatment than patients who did not. This association was not observed in autoantibody seronegative patients. Furthermore, serum BAFF levels below the threshold of <968.5 pg/mL at 6 months of TNFi treatment were strongly associated with good EULAR response in seropositive patients. 

B cells are receiving increasing attention with regard to their role as a pivotal driver in the pathophysiology of RA [4,5]. RA is characterised by the presence and high titres of certain autoantibodies. The development of autoantibodies is hypothesised to be explained by a failure of the immune system which renders it incapable of eliminating or controlling autoreactive B cells. This failure contributes to a break of B-cell tolerance, promoting the persistence of autoreactive B cells through defective central and peripheral B-cell development checkpoints [8]. In this regard, an excessive production of BAFF has been suggested to be a potential contributor to the breach of B-cell tolerance and the consequent development of autoantibodies [18,19]. In the context of RA, the presence of autoantibodies, such as RF and ACPA, has been associated with a higher degree of inflammation that leads to a more aggressive and joint-erosive disease course, and therefore worse prognosis owing to accelerated damage progression in these patients [11,12,13]. Along the same lines and supporting the role of B cells in RA pathophysiology, we showed that seropositive RA patients had higher baseline disease activity than seronegative patients of a similar disease duration.

Seropositivity has been associated with greater efficacy of non-TNFi biologicals such as rituximab or abatacept, but not TNFis [20]. However, other studies have found an influence of seropositivity on discontinuation and improvement in the HAQ (Health Assessment Questionnaire) and pain in patients with established RA treated with TNFis [12,21]. These controversies highlight the need to learn more about the underlying mechanisms between seropositive and seronegative patients, hence the interest of our research group in evaluating the role of BAFF serum levels in relation to autoantibody status and its association with the response to treatment with TNFis. Previous evidence has explored the association between serum or synovial fluid BAFF levels in patients with RA versus healthy controls [9,22]. In addition, the association of BAFF levels with autoantibody titres has been previously described [23]. Until the present work, no study has so clearly analysed serum levels of BAFF at baseline and 6 months after treatment with TNFis in patients with seropositive RA. We found that serum BAFF levels seem to only be relevant in seropositive patients. These findings indirectly reflect the implication of B cells in the pathogenesis and perpetuation of inflammation in patients with seropositive RA.

Currently, the role of B cells in the response to TNFis is still unclear and controversial. This controversy might be related to the variability within different RA cohorts and different criteria used to assess the clinical course and treatment response [24,25]. Furthermore, these different criteria can make the different studies incomparable. Therefore, due to this potential variability that may influence the results, we adjusted the analyses of our study by the variables that were associated more with good EULAR response in the univariable analysis. Leandro has deepened this controversy by suggesting that this variability could be due to the absence of a pattern in the B-cell compartment population in patients with RA [24]. Previous results from our research group and from other researchers have supported the hypothesis of the importance of B-cell dynamics in response to biological therapy [25,26,27]. Moreover, research from our group identified the role of BAFF in the development of immunogenicity and, therefore, in B-cell activation and its involvement in the response to TNFis [28]. Taking this information into account, and in order to investigate more deeply the role of BAFF in the response to TNFi therapy, we stratified our RA cohort by autoantibody positivity status. We found that both RF- and ACPA-positive patients with non-EULAR response after 6 months of TNFi treatment displayed an increase in serum BAFF concentrations during this period. With these findings, it could be hypothesised that in seropositive patients who are good responders, their disease is mediated by the chosen mechanism of action and could demonstrate less BAFF production by inflammatory cells and greater uptake by the specific receptor expressed on B cells [23]. This hypothesis has been reinforced by previous studies in which the proportion of B lymphocytes at baseline and 6 months after treatment with TNFis is different depending on the response to treatment [26,27].

Measurement of the inflammatory activity of the disease in patients with RA is complex. The established therapeutic goal is remission or low activity [29]. There are several integrated composite indices available that assess different parameters such as: painful and swollen joints, global assessment of the disease by the patient and/or the doctor, acute-phase reactants (ESR or CRP) [30]. These tools are very useful in clinical practice to monitor activity but have limitations such as: individual variability, sensitivity to change in interobserver joint count and lack of specificity of ESR or CRP [30,31,32]. Due to the aforementioned tools and the number of biological drugs with different mechanisms of action, there is a growing demand to define biomarkers that help predict or monitor response in clinical practice [33]. The problem is that many of those described to date are either not easily reproducible or have not shown clear clinical utility [33]. In relation to this, we found that 85% of RF-positive patients (or 81% of ACPA-positive patients) who had BAFF concentrations below 968 pg/mL were deemed good responders at 6 months. Moreover, seropositive RA patients who had BAFF concentrations below this threshold demonstrated more than a seven-fold increased probability for RF or four-fold for ACPA, and were classified as GRs. BAFF measurement is a reproducible technique and easy to perform in a laboratory. Therefore, it could be proposed as a more specific additional parameter to monitor response to treatment with TNFis in seropositive patients.

Considering the relevance of B-cell and BAFF levels in rheumatoid arthritis, an anti-BAFF monoclonal antibody such as belimumab would be expected to hold great promise. However, although it is a drug widely used in other pathologies such as systemic erythematous lupus [34], its development in RA failed. In clinical trials, belimumab was a drug well-tolerated in the treatment of RA. The ACR20 responses at week 24 were statistically significant, especially in patients with high disease activity, positive RF, no anti-TNF treatment experience and those who had failed methotrexate therapy [35]. However, belimumab failed to demonstrate significantly improved ACR50 and ACR70 responses in the single phase II clinical trial of RA [35]. On the other hand, rituximab, which is an anti-CD20 monoclonal antibody that completely depletes B cells, is a drug indicated in RA. Although there are no head-to-head studies comparing the two drugs, belimumab does not appear to be as effective as rituximab. Clinical studies have shown that rituximab has achieved greater response rates in the treatment of RA [36]. One possible explanation for these differences could be that rituximab induces complete depletion of B cells, whereas belimumab can only lower the B-cell count [35]. BAFF is a fundamental survival factor for transitional and mature B cells. Anti-BAFF therapy with belimumab can incompletely reduce circulating B-cell levels. Therefore, belimumab has the potential to modulate inflammatory and immune responses without totally losing the functions of B cells [35].

This study had some limitations, which should be kept in mind when interpreting the results. These included the low number of autoantibody-negative patients in our study population (RF: 32, 20%; ACPA: n = 24, 15%). Furthermore, regarding the population size, EULAR response was stratified into two categories based only on the EULAR response criteria, i.e., good response and non-response, thus overlooking the category of moderate response to ensure adequate power in statistical analyses. However, the minimal missingness of data may be considered a strength. Another limitation was the difference in baseline activity measured by DAS28 between patients according to autoantibody status. For this reason, we chose the EULAR response as the clinical outcome and adjusted the statistical analyses by baseline activity. Our future research agenda includes increasing the study population with the aim of exploring baseline BAFF levels as a predicting biomarker of response. 

In conclusion, the present study provides implications that serum BAFF may reflect the biological response to TNFi therapy in seropositive patients with RA and may therefore constitute a useful complement to clinical assessment. Further survey in other RA cohorts to confirm these results has merit, as does investigation of serum BAFF dynamics during therapy with other drug classes.

## Figures and Tables

**Table 1:** **Patients’ characteristics.** The table shows mean ± SD, median (IQR) or absolute number (percentage) for patients included (n = 158). The results are stratified by RF or ACPA seropositivity status. Significant statistical differences between non-responders and good responders are indicated as *p* < 0.05 (*), *p* < 0.01 (**) or *p* < 0.001 (***). Significant statistical differences between 0 months and 6 months are indicated as *p* < 0.05 (#). ACPA, anti-citrullinated peptide antibody; BAFF, B-cell-activating factor; csDMARDs, conventional synthetic disease-modifying anti-rheumatic drugs; DAS28, disease activity score-28; MDA, moderate disease activity; HAD, high disease activity; MTX, methotrexate; OD, other csDMARDs; RF, rheumatoid factor; TNFi, tumour necrosis factor inhibitor.

	All (n = 158)			RF Negative (n = 32)		RF Positive (n = 126)		ACPA Negative (n = 24)		ACPA Positive (n = 134)	
Patient Characteristics	Pooled	EULAR NR (n = 120; 76%)	EULAR GR (n = 38; 24%)	EULAR NR (n = 23; 72%)	EULAR GR (n = 9; 28%)	EULAR NR (n = 97; 77%)	EULAR GR (n = 29; 23%)	EULAR NR (n = 16; 67%)	EULAR GR (n = 8; 33%)	EULAR NR (n = 104; 78%)	EULAR GR (n = 30; 22%)
**Age (years)**	54 ± 14	54 ± 14	55 ± 16	54 ± 11	56 ± 18	54 ± 14	54 ± 16	49 ± 12	54 ± 17	55 ± 14	55 ± 16
**Female**	130 (82)	96 (80)	34 (89)	17 (74)	8 (89)	79 (81)	26 (90)	14 (87)	7 (87)	82 (79)	27 (90)
**Disease duration (years)**	8 (4–13)	9 (4–14)	7 (4–11)	5 (3–8)	6 (2–16)	11 (4–15)	7 (4–11) (*)	8 (4–11)	3 (1–14)	9 (4–14)	7 (5–11)
**RF positive**	126 (80)	97 (81)	29 (76)	0 (0)	0 (0)	97 (100)	29 (100)	5 (31)	4 (50)	92 (88)	25 (83)
**ACPA positive**	134 (85)	104 (87)	30 (79)	12 (52)	5 (56)	92 (95)	25 (86)	0 (0)	0 (0)	104 (100)	30 (100)
**Smokers**	74 (47)	57 (47)	17 (45)	10 (43)	5 (56)	47 (48)	12 (41)	8 (50)	4 (50)	49 (47)	13 (43)
**Body mass index (kg/m^2^)**	25.2 (21.8–29.7)	24.2 (22.7–26.2)	23.6 (21.4–26.2) (*)	24.7 (23.3–29.8)	23.5 (19.9–26.9)	26.5 (22.4–30.3)	23.6 (21.5–26.4)	26.7 (23.9–29.3)	23.4 (20.1–26.4)	26.2 (21.8–30.4)	23.8 (21.5–26.3)
**Baseline DAS28**	5.1 ± 1.3	5.2 ± 1.4	4.8 ± 0.8 (*)	4.4 ± 1.2	4.4 ± 0.6	5.5 ± 1.4	5.0 ± 0.6 (*)	4.6 ± 1.1	4.6 ± 0.9	5.4 ± 1.4	4.9 ± 0.8 (*)
MDA (DAS28: 3.2–5.1)	78 (49)	52 (43)	26 (68) (**)	17 (74)	8 (89)	35 (36)	18 (62) (*)	11 (69)	6 (75)	41 (39)	20 (67) (***)
HDA (DAS28 > 5.1)	80 (51)	68 (57)	12 (32) (**)	6 (26)	1 (11)	62 (64)	11 (38) (*)	5 (31)	2 (25)	63 (61)	10 (33) (***)
**Previous TNFi**	18 (11)	16 (13)	2 (5)	4 (17)	0 (0)	12 (12)	2 (7)	4 (25)	1 (12)	12 (11)	1 (3)
**Concomitant csDMARDs**	144 (91)	108 (90)	36 (95)	21 (91)	8 (89)	87 (90)	28 (97)	14 (87)	7 (87)	94 (90)	29 (97)
MTX [±OD]	104 (66)	78 (65)	26 (68)	13 (56)	5 (56)	65 (67)	21 (72)	8 (50)	6 (75)	70 (67)	20 (67)
MTX dose (mg/week)	20.0 (15.0–25.0)	20.0 (15.0–25.0)	20.0 (12.5–22.5)	20.0 (20.0–25.0)	20.0 (10.0–22.5)	20.0 (13.7–25.0)	20.0 (12.5–22.5)	20.0 (20.0–25.0)	18.7 (15.0–20.6)	20.0 (13.4–25.0)	20.0 (12.5–24.4)
Only OD	40 (25)	30 (25)	10 (26)	8 (35)	3 (33)	22 (23)	7 (24)	6 (37)	1 (12)	24 (23)	9 (30)
**Prednisone**	83 (52)	66 (55)	17 (45)	13 (56)	4 (44)	53 (55)	13 (45)	10 (62)	3 (37)	56 (54)	14 (47)
Prednisone dose (mg/day)	5.0 (0.0–5.0)	5.0 (0.0–5.0)	0.0 (0.0–5.0)	5.0 (0.0–5.0)	0.0 (0.0–5.0)	5.0 (0.0–5.0)	0.0 (0.0–5.0)	5.0 (2.5–6.2)	0.0 (0.0–5.0) (*)	5.0 (0.0–5.0)	0.0 (0.0–5.0)
**Baseline Serum BAFF (pg/mL)**	844 (686–1054)	866 (701–1060)	754 (622–922) (*)	834 (706–1120)	854 (765–990)	870 (691–1060)	746 (616–865) (*)	792 (732–963)	756 (625–899)	876 (684–1020)	754 (622–891)
**6 months Serum BAFF (pg/mL)**	890 (722–1074) (**#**)	917 (792–1044) (**#**)	793 (715–956) (*)	846 (670–1105)	845 (713–1011)	980 (795–1170) (**#**)	787 (715–922) (*)	856 (697–992)	758 (715–1090)	955 (808–1176) (**#**)	793 (712–956) (**)

**Table 2:** **Association between patient characteristics and EULAR response at 6 months (univariable and multivariable analyses), stratified by RF or ACPA seropositivity.** The univariable and adjusted-multivariable logistic regression analyses were performed. Multivariable analyses were adjusted by variables with *p* < 0.1 at the univariable tests. Odds ratio (OR) and 95% confidence interval (CI) were calculated. Significant statistical differences are noted in bold. *p*-value < 0.05 was considered statistically significant. ACPA, anti-citrullinated peptide antibody; BAFF, B-cell-activating factor; csDMARDs, conventional synthetic disease-modifying anti-rheumatic drugs; DAS28, disease activity score-28; MTX, methotrexate; OD, other csDMARDs; RF, rheumatoid factor; TNFi, tumour necrosis factor inhibitor.

	RF Positive (n = 126)						ACPA Positive (n = 134)					
	Univariable Analysis			Multivariable Analysis			Univariable Analysis			Multivariable Analysis		
Patient Characteristics	OR	95% CI	*p*-Value	OR	95% CI	*p*-Value	OR	95% CI	*p*-Value	OR	95% CI	*p*-Value
** Age (years)**	1.00	0.97–1.03	1.0					0.97–1.03	0.9			
** Female**	1.97	0.54–7.25	0.3				2.41	0.67–8.70	0.2			
** Disease duration (years)**	0.93	0.86–0.99	**0.03**	0.90	0.83–0.98	**0.02**	0.95	0.90–1.01	0.1			
** RF positive**	-	-	-				0.65	0.21–2.02	0.5			
** ACPA positive**	0.34	0.08–1.36	0.1				-	-	-			
** Smokers**	0.75	0.32–1.73	0.5				0.86	0.38–1.95	0.7			
** Body mass index (kg/m^2^)**	0.92	0.84–1.00	0.06	0.90	0.81–1.00	0.06	0.92	0.84–1.01	0.07	0.90	0.81–1.00	0.05
** DAS28**	0.74	0.54–1.03	0.07	0.80	0.52–1.23	0.3	0.74	0.53–1.04	0.08	0.78	0.53–1.14	0.2
** Previous TNFi**	0.52	0.11–2.49	0.4				0.26	0.03–2.12	0.2			
** Concomitant csDMARDs**	3.22	0.39–26.26	0.3				3.08	0.38–25.13	0.3			
MTX [±OD]	1.29	0.52–3.23	0.6				0.97	0.41–2.30	0.9			
MTX dose (mg/week)	1.00	0.92–1.09	1.0				0.98	0.91–1.07	0.7			
Only OD	1.08	0.41–2.87	0.9				1.43	0.58–3.53	0.4			
** Prednisone**	0.67	0.29–1.55	0.3				0.75	0.33–1.69	0.5			
Prednisone dose (mg/day)	0.90	0.78–1.05	0.2				0.94	0.81–1.09	0.4			
** 6 months Serum BAFF < 968.5 pg/mL**	5.95	1.87–18.88	**0.002**	7.94	2.32–27.22	**0.001**	4.40	1.52–12.77	**0.006**	4.74	1.58–14.23	**0.006**

## Data Availability

The datasets generated and/or analysed during the current study are available from the corresponding author on reasonable request.

## Appendix A

**Table A1:** Generalised estimating equations (GEEs) model. The response variable means were estimated and compared by least squares between the different levels of each categorical independent variable. ACPA, anti-citrullinated peptide antibody; BAFF, B-cell-activating factor; csDMARDs, conventional synthetic disease-modifying anti-rheumatic drugs; DAS28, disease activity score-28; MTX, methotrexate; OD, other csDMARDs; RF, rheumatoid factor.

Outcome Variable in GEE Model	Independent Variables	Regression Coefficient (ß) 95% CI	*p*-Value
**Serum BAFF (pg/mL)**	Follow up time	−0.46 (−1.05, 0.12)	0.1
	Female	0.53 (−0.85, 1.92)	0.4
	**Age (years)**	0.04 (0.006, 0.08)	0.02
	** Age*time**	0.02 (−0.02, 0.07)	0.3
	Disease duration (years)	0.03 (−0.02, 0.09)	0.3
	RF positive	0.40 (−1.15, 1.95)	0.6
	**ACPA positive**	−1.04 (−2.05, −0.03)	0.04
	** ACPA positive*time**	−0.55 (−1.45, 0.35)	0.6
	Smokers	0.58 (−0.47, 1.62)	0.3
	Body mass index (kg/m^2^)	−0.06 (−0.16, 0.05)	0.3
	DAS28	−0.16 (−0.38, 0.04)	0.1
	Concomitant csDMARDs	0.19 (−1.32, 1.71)	0.8
	MTX [±OD]	0.07 (−1.17, 1.31)	0.9
	MTX dose (mg/week)	−0.03 (−0.48, 0.63)	0.6
	Only OD	−0.006 (−1.47, 1.46)	1.0
	Prednisone	0.39 (−0.71, 1.49)	0.5

## References

[1] Souto A., Maneiro J.R., Gómez-Reino J.J. (2016). Rate of discontinuation and drug survival of biologic therapies in rheumatoid arthritis: A systematic review and meta-analysis of drug registries and health care databases. Rheumatology.

[2] Smolen J.S., Landewé R., Bijlsma J., Burmester G., Chatzidionysiou K., Dougados M., Ramiro S., Voshaar M., van Vollenhoven R., Aletaha D. (2017). EULAR recommendations for the management of rheumatoid arthritis with synthetic and biological disease-modifying antirheumatic drugs: 2016 update. Ann. Rheum. Dis..

[3] Xie X., Li F., Li S., Tian J., Chen J.-W., Du J.-F., Mao N., Chen J. (2018). Application of omics in predicting anti-TNF efficacy in rheumatoid arthritis. Clin. Rheumatol..

[4] Gravallese E.M., Robinson W.H. (2020). PRIME Time in Rheumatoid Arthritis. N. Engl. J. Med..

[5] Orange D.E., Yao V., Sawicka K., Fak J., Frank M.O., Parveen S., Blachere N.E., Hale C., Zhang F., Raychaudhuri S. (2020). RNA Identification of PRIME Cells Predicting Rheumatoid Arthritis Flares. N. Engl. J. Med..

[6] Kreuzaler M., Rauch M., Salzer U., Birmelin J., Rizzi M., Grimbacher B., Plebani A., Lougaris V., Quinti I., Thon V. (2012). Soluble BAFF Levels Inversely Correlate with Peripheral B Cell Numbers and the Expression of BAFF Receptors. J. Immunol..

[7] Pers J.-O., Daridon C., Devauchelle V., Jousse S., Saraux A., Jamin C., Youinou P. (2005). BAFF Overexpression Is Associated with Autoantibody Production in Autoimmune Diseases. Ann. N. Y. Acad. Sci..

[8] Pouw J.N., Leijten E.F.A., van Laar J.M., Boes M. (2021). Revisiting B cell tolerance and autoantibodies in seropositive and seronegative autoimmune rheumatic disease (AIRD). Clin. Exp. Immunol..

[9] Bosello S., Youinou P., Daridon C., Tolusso B., Bendaoud B., Pietrapertosa D., Morelli A., Ferraccioli G. (2008). Concentrations of BAFF correlate with autoantibody levels, clinical disease activity, and response to treatment in early rheumatoid arthritis. J. Rheumatol..

[10] Giltiay N.V., Chappell C.P., Clark E.A. (2012). B-cell selection and the development of autoantibodies. Arthritis Res. Ther..

[11] Pongratz G., Frieser R., Brinks R., Schneider M., Hartung W., Fleck M., Ehrenstein B. (2020). Association between autoantibody level and disease activity in rheumatoid arthritis is dependent on baseline inflammation. Clin. Exp. Rheumatol..

[12] Ogawa Y., Takahashi N., Kaneko A., Hirano Y., Kanayama Y., Yabe Y., Oguchi T., Fujibayashi T., Takagi H., Hanabayashi M. (2019). Association between seropositivity and discontinuation of tumor necrosis factor inhibitors due to ineffectiveness in rheumatoid arthritis. Clin. Rheumatol..

[13] Aletaha D., Alasti F., Smolen J.S. (2013). Rheumatoid factor determines structural progression of rheumatoid arthritis dependent and independent of disease activity. Ann. Rheum. Dis..

[14] Novella-Navarro M., Plasencia C., Tornero C., Navarro-Compán V., Cabrera-Alarcón J.L., Peiteado-López D., Nuño L., Monjo-Henry I., Franco-Gómez K., Villalba A. (2020). Clinical predictors of multiple failure to biological therapy in patients with rheumatoid arthritis. Arthritis Res. Ther..

[15] Aletaha D., Neogi T., Silman A.J., Funovits J., Felson D.T., Bingham C.O., Birnbaum N.S., Burmester G.R., Bykerk V.P., Cohen M.D. (2010). 2010 rheumatoid arthritis classification criteria: An American College of Rheumatology/European League against Rheumatism collaborative initiative. Arthritis Rheum..

[16] Sanmartí R., García-Rodríguez S., Álvaro-Gracia J.M., Andreu J.L., Balsa A., Cáliz R., Fernández-Nebro A., Ferraz-Amaro I., Gómez-Reino J.J., González-Álvaro I. (2015). 2014 Update of the Consensus Statement of the Spanish Society of Rheumatology on the Use of Biological Therapies in Rheumatoid Arthritis. Reumatol. Clin..

[17] van Gestel A.M., Prevoo M.L., van’t Hof M.A., van Rijswijk M.H., van de Putte L.B., van Riel P.L. (1996). Development and validation of the European league against rheumatism response criteria for rheumatoid arthritis: Comparison with the preliminary American College of Rheumatology and the World Health Organization/International League against Rheumatism Criteria. Arthritis Rheum..

[18] Thien M., Phan T., Gardam S., Amesbury M., Basten A., Mackay F., Brink R. (2004). Excess BAFF Rescues Self-Reactive B Cells from Peripheral Deletion and Allows Them to Enter Forbidden Follicular and Marginal Zone Niches. Immunity.

[19] Rose W.A., Okragly A.J., Hu N.N., Daniels M.R., Martin A.P., Koh Y.T., Kikly K., Benschop R.J. (2018). Interleukin-33 Contributes toward Loss of Tolerance by Promoting B-Cell-Activating Factor of the Tumor-Necrosis-Factor Family (BAFF)-Dependent Autoantibody Production. Front. Immunol..

[20] Courvoisier D.S., Chatzidionysiou K., Mongin D., Lauper K., Mariette X., Morel J., Gottenberg J.-E., Bergstra S.A., Suarez M.P., Codreanu C. (2021). The impact of seropositivity on the effectiveness of biologic anti-rheumatic agents: Results from a collaboration of 16 registries. Rheumatology.

[21] Aberumand B., Barra L., Cao Y., Le Riche N., Thompson A.E., Rohekar G., Rohekar S., Bonner A., Pope J.E. (2014). Response to Tumor Necrosis Factor Inhibitors in Rheumatoid Arthritis for Function and Pain is Affected by Rheumatoid Factor. Open Rheumatol. J..

[22] Cheema G.S., Roschke V., Hilbert D.M., Stohl W. (2001). Elevated serum B lymphocyte stimulator levels in patients with systemic immune-based rheumatic diseases. Arthritis Rheum..

[23] Shabgah A.G., Shariati-Sarabi Z., Tavakkol-Afshari J., Mohammadi M. (2019). The role of BAFF and APRIL in rheumatoid arthritis. J. Cell. Physiol..

[24] Leandro M.J. (2009). Anti-tumour necrosis factor therapy and B cells in rheumatoid arthritis. Arthritis Res. Ther..

[25] Daien C.I., Gailhac S., Mura T., Combe B., Hahne M., Morel J. (2014). High levels of memory B cells are associated with response to a first tumor necrosis factor inhibitor in patients with rheumatoid arthritis in a longitudinal prospective study. Arthritis Res. Ther..

[26] Rodríguez-Martín E., Nieto-Gañán I., Hernández-Breijo B., Sobrino C., García-Hoz C., Bachiller J., Martínez-Feito A., Navarro-Compán V., Lapuente-Suanzes P., Bonilla G. (2020). Blood Lymphocyte Subsets for Early Identification of Non-Remission to TNF Inhibitors in Rheumatoid Arthritis. Front. Immunol..

[27] Hernández-Breijo B., Plasencia-Rodríguez C., Navarro-Compán V., García-Hoz C., Nieto-Gañán I., Sobrino C., Bachiller-Corral J., Díaz-Almirón M., Martínez-Feito A., Jurado T. (2021). Remission Induced by TNF Inhibitors Plus Methotrexate is Associated with Changes in Peripheral Naïve B Cells in Patients with Rheumatoid Arthritis. Front. Med..

[28] Hernández-Breijo B., Navarro-Compán V., Plasencia-Rodríguez C., Parodis I., Gehin J.E., Martínez-Feito A., Novella-Navarro M., Mezcua A., Warren D.J., Nozal P. (2021). BAFF predicts immunogenicity in older patients with rheumatoid arthritis treated with TNF inhibitors. Sci. Rep..

[29] Smolen J.S., Landewé R.B.M., Bijlsma J.W.J., Burmester G.R., Dougados M., Kerschbaumer A., McInnes I.B., Sepriano A., van Vollenhoven R.F., de Wit M. (2020). EULAR recommendations for the management of rheumatoid arthritis with synthetic and biological disease-modifying antirheumatic drugs: 2019 update. Ann. Rheum. Dis..

[30] Chandrashekara S., Sachin S. (2012). Measures in rheumatoid arthritis: Are we measuring too many parameters. Int. J. Rheum. Dis..

[31] Pincus T. (2008). Limitations of a quantitative swollen and tender joint count to assess and monitor patients with rheumatoid arthritis. Bull. NYU Hosp. Jt. Dis..

[32] Salaffi F., Filippucci E., Carotti M., Naredo E., Meenagh G., Ciapetti A., Savic V., Grassi W. (2008). Inter-observer agreement of standard joint counts in early rheumatoid arthritis: A comparison with grey scale ultrasonography a preliminary study. Rheumatology.

[33] Takeuchi T. (2018). Biomarkers as a treatment guide in rheumatoid arthritis. Clin. Immunol..

[34] Navarra S.V., Guzmán R.M., Gallacher A.E., Hall S., Levy R.A., Jimenez R.E., Li E.K.-M., Thomas M., Kim H.-Y., León M.G. (2011). Efficacy and safety of belimumab in patients with active systemic lupus erythematosus: A randomised, placebo-controlled, phase 3 trial. Lancet.

[35] Jin X., Ding C. (2013). Belimumab—An anti-BLyS human monoclonal antibody for rheumatoid arthritis. Expert Opin. Biol. Ther..

[36] Edwards J.C., Szczepanski L., Szechinski J., Filipowicz-Sosnowska A., Emery P., Close D.R., Stevens R.M., Shaw T. (2004). Efficacy of B-cell-targeted therapy with rituximab in patients with rheumatoid arthritis. N. Engl. J. Med..

